# Timolol 0.1% in Glaucomatous Patients: Efficacy, Tolerance, and Quality of Life

**DOI:** 10.1155/2019/4146124

**Published:** 2019-05-02

**Authors:** Letizia Negri, Antonio Ferreras, Michele Iester

**Affiliations:** ^1^Anatomical-Clinical Laboratory for Functional Diagnosis and Treatment of Glaucoma and Neuro-ophthalmology, Eye Clinic, DiNOGMI, University of Genoa, Genoa, Italy; ^2^Department of Ophthalmology, IIS-Aragon, Miguel Servet University Hospital, University of Zaragoza, Zaragoza, Spain

## Abstract

Glaucoma is a progressive, chronic optic neuropathy characterized by a typical visual field defects. Four main classes of topical medication are actually available on the market: beta-blockers, prostaglandins, alpha2-agonists, and topical carbonic anhydrase inhibitor to treat intraocular pressure (IOP). The aim of this review is to outline the efficacy of timolol and to evaluate the impact of this treatment on patients' quality of life. Among beta-blockers, timolol is most used at three different concentrations: 0.1%, 0.25%, and 0.5%. While the first one is a gel, the other two products are solution. Timolol has few topical side effects, while it has some important systemic side effects on the cardiac and respiratory systems. The balance between efficacy and safety is always the main aspect to care patients. Because of the less efficacy of timolol 0.1% solution, the possibility to use carbomers as vehicle in the gel drops helped timolol 0.1 to be used in clinics, extending the time contact between the active ingredient and the surface of the cornea. Using preservative-free timolol 0.1 for treatment, IOP was at the same level of the other beta-blockers at higher concentration, but it was better tolerated. Preservative-free treatment improved the quality of life reducing dry-eye like symptoms; furthermore, the presence of an artificial tear in the medication bottle could help adherence. The once daily dosing improves compliance.

## 1. Introduction

The intraocular pressure (IOP) is determined by the balance of aqueous humor production and drainage. While production occurs via the ciliary body, drainage occurs predominantly (50–75%) through the trabecular meshwork and secondarily (25–50%) through the ciliary muscle, supraciliary space, suprachoroidal space, and sclera (uveoscleral outflow). [1–3] Within the general population, the distribution of “normal” IOP is almost Gaussian except for a slight skew toward higher pressures. The mean value is approximately 15 mmHg, and two standard deviations to either side of the mean give a “normal” range of approximately 10–20 mmHg. However, the concept of normal or abnormal IOP value is mainly theoretical except for the high values, and any IOP value could be pathological for a given optic nerve head (ONH) [1–3]. 24 hour variations in IOP are mainly due to variation in aqueous humor production [4], and this process is principally regulated by sympathetic system; in fact, catecholamines that are secreted by adrenal medulla induce the aqueous humor production following a 24 hour cycle, and their level monitored with urine secretion falls down during the sleep phase [5].

In healthy subjects, IOP variations are characterized by a diurnal average that is lower than the nocturnal; in this case, the 24 hour-IOP curve takes the shape of a sine curve and the dimension of variation is around 3–6 mmHg; in particular, clearance of aqueous humor during sleep is reduced by half compared to the first hour after waking [5, 6]. On the contrary, in glaucomatous patients, the 24 hour variations can reach up to 18 mmHg with the inversion of the circadian rhythm, with the average value higher during the day with a characteristic peak in the morning and a nocturnal IOP reduction lower than in healthy subjects [4–9].

Under the clinical term of glaucoma, there are different conditions with the common feature of an optic neuropathy characterized by a distinctive loss of retinal nerve fiber layer (RNFL) and optic nerve head (ONH) defects. The loss of retinal ganglion cells can lead to an irreversible loss of visual field, usually beginning paracentrally, but becoming complete if the disease is uncontrolled. The only risk factor that we can modify is the IOP, and IOP modification is possible by using topical drops [10].

Four main classes of topical medication are actually available in the market: beta-blockers, prostaglandins, alpha2-agonists, and topical carbonic anhydrase inhibitor (CAI). Among these four classes, beta-blockers are the oldest one. Beta-blockers are still one of the classes most used by glaucoma patients, whether in monotherapy or combined therapy. Over 40% of glaucoma or hypertensive patients are on beta-blockers, and nearly one out of every two new patients receives a beta-blocker as a first line therapy, usually timolol.

### 1.1. Beta-Blockers

They lower IOP level acting on beta-adrenergic receptors located on the nonpigmented ciliary epithelium and on vessels, thereby limiting active transport of aqueous humor, thus reducing the production; in long-term treatment, the secretion can fall by 40 to about 50 %. Also, vascular mechanism that reduces the passive generation of aqueous by ultrafiltration may be involved. Timolol's blocking action is powerful, nonselective *β*1-*β*2, lipophilic (essential property for penetrating the cornea, except for carteolol) without any type of membrane-stabilizing activity [11].

In the market, three different concentrations of topical timolol are available, which are 0.1%, 0.25%, and 0.5%. While the first one is a gel, the other two products are solution. Timolol has few topical side effects, while it has some important systemic side effects on the cardiac and respiratory systems. The balance between efficacy and safety is always the main aspect to care patients.

Because of the less efficacy of timolol 0.1% solution, the possibility to use carbomers as vehicle in the gel drops helped timolol 0.1 to be used in clinics, extending the contact time between the active ingredient and the surface of the cornea. Carbomers are polymers created by cross-linking acrylic acid with ethers in the presence of an organic solvent.

These macromolecules create a three-dimensional network able to keep a huge quantity of water in their mesh (about 1000 times their dry volume). For this property, an aqueous, stable, transparent, colorless, nonsticky gel is so required.

Carbomers are particularly appropriate for ophthalmic use for some peculiar properties:

- Their viscosity decreases very rapidly, so they can cover entirely the surface of the eye
- They instantly restore when eyelids are open for their rheological properties
- They do not run when placed on a slope, and this is particularly useful for a well coverage of the entire eye
- Their dissolution is very gradual because they stabilize with mucous layer glycoproteins of the tear film

The principal reason for prolonged ocular contact time is represented by carbomer's mucoadhesive properties while viscosity plays only a minor role [12]. Carbopol 974P is the carbomer used for timolol 0.1% gel (TimoGel 0.1%, Thea), in which the solvent is ethyl acetate instead of benzene commonly used in other ophthalmic gels. The clearance of an ophthalmic solution measured by radioisotopes depends only in part on viscosity; as a matter of fact, carbopol and polyvinyl alcohol (PVA) have the same viscosity, but residual corneal activity after one hour from instillation is higher for the first one, but both agents are however superior to a simple solution [13].

The viscosity of pure carbomer often causes blurred vision for several minutes after administration; that is why TimoGel 0.1% is used, which is a gel obtained by the combination of PVA and carbopol that spreads quickly over the surface of the eye, also maintaining the mucoadhesive properties of a carbomer. This structure is also used to vehicle the gradual release of timolol in the eye [12–14].

Carbomers have the benefit of staying for a prolonged time over cornea and conjunctiva surface, while other liquid gels have a residence time comparable to solutions. It is widely demonstrated through scintigraphic studies of corneal contact time that a standard saline solution disappears in less than 20 minutes from the surface of the eye, while a mucoadhesive gel stays for over 40 minutes after the instillation at an acceptable quantity [14].

Several studies have already confirmed that use of carbomers have become the first-choice therapy for patients who suffer from dry eye disease increasing tear break-up time (BUT) and improving Schrimer's test results, also in healthy patients [15–17].

Carbomers are often used as vehicle the gradual release of various active agents for instance 2% pilocarpin (12–14). The concentration of an active agent combined with a carbomer gel is significantly higher when the same ingredient is instead combined with a standard solution [18].

### 1.2. Ocular and Systemic Pharmacodynamics of Timolol Gel

The elimination half life of timolol on the eye surface is about 100 times longer when the vehicle is a carbomer gel than when it is a simple saline solution and 10 times longer than a viscous gel of hydroxyethylcellulose; in this way, carbomers extend bioavailability and increase absorption of active agents [19].

It is well demonstrated in animal studies that measurable levels of timolol are found one hour after administrating 0.1% gel formulation instead of 0.5% timolol solution that disappears in about half an hour [20]; in fact, local penetration of gel formulation is 2.6 to 3.1 times greater than the 0.5% timolol solution [19].

A study performed on rabbits showed that a gel formulation with half concentration of timolol (0.05%) had peak concentration in the aqueous humor that was three times higher than a conventional 0.1% timolol solution [21]. In addition to this, when the beta-blocker arrives in the aqueous humor, it spreads on the tissue of the anterior segment and in particular in the granules of melanin in the ciliary body, allowing a gradual release of the active principal ingredient and improving its bioavailability in the receptor sites [11]. In humans, when compared to a 0.5% timolol solution, it is demonstrated that TimoGel 0.1% reduces the dose and the frequency of administration while maintaining the same therapeutic efficacy [22–37].

The systemic absorbance of timolol when administered by topical ocular instillation is rapid and occurs principally through the nasopharyngeal tract [19]. The feasible adverse events are related to the dose of exposition and to the plasma concentration of beta-blockers, this possibility increases rapidly after the threshold of 200 pg/ml [38].

When a single dose of 0.2 mg of timolol is administered either intravenously or by topical ocular instillation, plasma concentration is reached immediately with intravenous administration, whereas for topical use, plasma concentration is reached about 24 minutes after instillation of the topical agent, and its peak plasma concentration is about 4 times lower than that obtained with intravenous administration [38].

In comparison to ocular instillation, oral administration of a single dose of 20 mg of timolol causes a concentration 72 times higher than that produced by the common ocular topical preparation [39]. While the absorption of topical timolol into the systemic circle is lower than that in oral assumption, the mean systemic absorbance of timolol in ocular instillation is about 78%, so the systemic side effects are less evident than those with oral administration but still present [40, 41].

Timolol in gel formulation permits a lower concentration and a slower release of active agents to obtain systemic kinetics similar to those of oral administration. A French study was performed to compare systemic absorption of three types of formulation of timolol eye drops: 0.1% timolol maleate gel, 0.5% timolol aqueous solution, and 0.5% timolol maleate gel [42].

Maximum plasmatic concentration (*C*_max_) was significantly (*p*=0.0003) lower with timolol 0.1% gel than that with timolol 0.5% aqueous solution after both the first instillation and two weeks of treatment. Its mean plasma concentrations did not exceed 0.10 ng/mL after instillation of timolol 0.1% gel at either time-point measured, but they reached 0.80 ng/mL and 0.66 ng/mL after two weeks of treatment with timolol 0.5% aqueous solution and 0.5% gel, respectively. The areas under the concentration-time curve (AUC) values were 15 to 38 folds higher after ocular administration of 0.5% preparation than those after administration of 0.1% gel. After timolol 0.5% aqueous solution treatment, mean half-life (*t* 1/2) was approximately 3 h 30 m, while it was approximately 4 h 30 m after timolol 0.5% gel; for timolol 0.1% gel, *t* 1/2 was not obtained because too many values were below the lower limit of assessment [42]. After instillation of 0.1% maleate gel, its *C*_max_ was reduced by almost 90% compared to concentrations obtained after instillation of both 0.5% aqueous solution and 0.5% gel. Between 0 and 12 hours postadministration, AUCs were also reduced by up to 93 to 98%. In all patients, after 8 h or 24 h 0.1% from maleate gel instillation, timolol could not be quantified in the plasma [42].

The three essential factors to consider when prescribing a glaucoma therapy are efficacy, tolerance, and quality of life of patients.

## 2. Clinical Efficacy

Several studies have already demonstrated the efficacy of timolol in treating glaucomatous patients; in fact, in long-term therapy, IOP falls down from 27 up to 35% of its original value; the mechanism of action is essentially due to the reduction of aqueous humor production more for timolol than that for other beta-blocker agents such as carteolol or betaxolol [23, 24, 41].

### 2.1. Efficacy of a Single Dose of 0.1% Timolol Gel in Healthy Volunteers

Two clinical studies comparing the 0.1% timolol gel to 0.5% timolol solution in healthy volunteers have shown that there was a IOP reduction of 30% at peak compared to the baseline, with an equivalent decreasing curve for both products [31, 43]. The similar efficacy profile of IOP-lowering effect of 0.1% timolol in gel compared to a 0.5% timolol solution in healthy volunteers was confirmed also in a recent French study [42].

### 2.2. Timolol 0.1% Gel vs Timolol 0.5% Solution in Glaucoma Patients

A prospective, randomized, cross-over study investigated the circadian intraocular pressure and blood pressure (BP) reduction obtained with timolol maleate 0.5% solution administered twice daily versus timolol 0.1% in gel-forming carbomer administered in the morning in patients with primary open-angle glaucoma (POAG). Both timolol formulations showed the same 24-hour-IOP efficacy profile with a once-a-day and well-tolerated administration for the formulation in gel [27].

Furthermore, in different studies, when administered in monotherapy, 0.1% timolol gel reduces IOP by 27% (*p* < 0.001) [22–33], comparable to a conventional 0.5% timolol solution and superior to a 0.1% timolol solution (*p* < 0.001) [37].

Therapeutic efficacy of 0.1% timolol gel in patients with POAG or Ocular Hypertension (OH).

All the published studies on the efficacy of 0.1% timolol gel have demonstrated an efficacy at least comparable to benchmark treatments [22, 32–37] (Table 1).

### 2.3. Efficacy of 0.1% Timolol Gel as a Substitution for a Previous Therapy

A recent study confirmed that the use of preservative-free gel of timolol 0.1% once daily maintained the efficacy on IOP and reduced signs and symptoms in almost all glaucomatous patients treated by preserved latanoprost with signs of intolerance. On the basis of all these experimental and clinical reports, it should be recommended to use benzalkonium-free eye drops whenever possible, especially in patients with prolonged treatments, in those suffering from preexisting or concomitant ocular surface diseases, and those experiencing side effects related to the ocular surface [44, 45].

### 2.4. Efficacy of Timolol as an Adjunctive Therapy

Timolol works in synergy with the other classes of drugs currently prescribed in glaucoma, namely, CAIs and prostaglandins. Prostaglandin agonists (latanoprost, bimatoprost, and travoprost) act by increasing uveoscleral outflow. Their combination with beta-blockers has an additional effect on IOP [29, 44–46]. The combination of 0.1% timolol gel and latanoprost results in an overall IOP reduction of 38% (*p* < 0.012). The administration of a dual therapy of timolol and travoprost in patients with an average pretreatment IOP of 25 to 27 mmHg results in a reduction to 16-17 mmHg, an average reduction of 8 to 10 mmHg (32 to 38%) [41].

After two weeks on timolol or latanoprost alone, IOP was reduced to around 18-19 mmHg; the combination of timolol and latanoprost resulted in an additional significant IOP reduction (*p* < 0.01) of 13 to 14% in comparison to each treatment used alone. This allowed an IOP of 15 to 16 mmHg to be attained [29], while the combination of bimatoprost or travoprost with timolol results in an additional IOP reduction of around 5 to 7 mmHg [45, 46].

Topical CAIs reduce production of aqueous humor by directly inhibiting carbonic anhydrase in ciliary processes. Their combination with timolol results in an additional reduction of around 17% for dorzolamide and brinzolamide [47].

### 2.5. Timogel and Fixed Combination vs Unfixed Combination

Reducing the number of drugs is known to promote compliance and generally improve local tolerance by decreasing the quantity of preservative. Moreover, the successive administration of two lots of eye drops leads to the second medication washing the first, with a dilution of 45% if patients wait only 30 seconds and of 17% if they wait two minutes. The ideal interval of at least five minutes is rarely respected [48]. These considerations have led to the development and launch of fixed combinations, particularly with timolol, as a way of avoiding the disadvantages of dual therapies. However, in order to act on the morning IOP peak, timolol needs to be administered in the morning as its peak efficacy is reached after two or three hours. Prostaglandin analogues on the other hand attain their maximum efficacy in seven to eight hours and are more effective when administered in the evening.

Moreover, prostaglandins are normally administered once a day, so the same has to apply for a fixed combination. This limits doses of timolol, which is usually instilled twice daily. Two clinical studies comparing the effect of a latanoprost/timolol fixed combination versus latanoprost and timolol gel-forming solution unfixed combination in glaucomatous patients have demonstrated that the concomitant solution leads to a larger additional IOP reduction and lower daytime IOP levels as compared with the fixed combination, although the fixed combination has some potential advantages, such as improved compliance, better adherence, and less exposure to preservatives. The potential benefits of fixed combination therapy with latanoprost/timolol need to be considered against the reduced efficacy compared with unfixed concomitant therapy [48, 49]. These studies confirmed that fixed combinations are slightly less effective than concomitant administration of their components; in particular, as regards timolol and prostaglandin agonists, a significant difference in IOP efficacy (1.1 mmHg on average) has been evidenced, which favors the concomitant administration of timolol and latanoprost. Overall, the administration of these two products once daily could appear preferable: administering prostaglandin in the evening and 0.1% timolol gel in the morning with a better efficacy.

### 2.6. Timolol 0.1% Gel in Ocular Surgery

The most frequent complication after ocular surgery is the increase of IOP level that frequently occurs for various causes including inflammation, pigment dispersion, viscosurgical device, greater vascular permeability, or intravitreal injection (IVI) of anti-VEGF active agents [50, 51].

In a prospective, double-blinded, randomized study, 70 patients who underwent uncomplicated cataract surgery with phacoemulsification and intraocular lens implantation were included and divided into three different subgroups: 25 patients received a single instillation of timolol 0.1% gel, 20 a single instillation of timolol 0.5% eyedrops, and 25 no treatment. In conclusion, timolol 0.1% gel was as effective as timolol 0.5% eyedrops in reducing IOP and in limiting the occurrence of IOP spikes for up to 24 hours after phacoemulsification [50].

In another prospective study, all the patients who underwent an IVI of ranibizumab were randomly divided into three groups: 50 were not treated with timolol before the IVI, 50 received an instillation of timolol 0.1% gel the evening before the procedure, and 50 received an instillation of timolol 0.1% gel 2 hours before the IVI. From the results, IOP spikes are a frequent complication of anti-VEGF IVIs, the incidence of hypertensive IOP 5 minutes after IVI was significantly less in patients who received timolol 0.1% gel 2 hours before IVI, who also benefited from significantly greater protection against spikes >40 mmHg. These findings suggest that the routine prophylactic use of timolol 0.1% gel 2 hours before IVI is a safe and effective means of preventing IOP spikes, reducing the need for emergency procedures and preserving the health of the optic nerve [51].

## 3. Tolerance

Medical education has held that beta-blocker agents should be avoided in patients with congestive heart failure and symptomatic bradycardia as well as more advanced degrees of heart block; however, they have long played an integral role in the management of cardiovascular disease. It has been shown how beta-blockers benefit patients with compensated heart failure, and the American College of Cardiology recommends that all patients with depressed left ventricular function, when clinically stable, begin beta-blocker therapy, regardless of whether or not they had a myocardial infarction [5–9,11]. Furthermore, all patients who have suffered myocardial infarction should undergo beta-blocker therapy for at least 3 years [52].

We have to remember that the literature fails to support many of the traditionally cited negative effects of beta-blockers (i.e., intermittent claudication, prolonged hypoglycemia in type II diabetics, or worsening symptoms of peripheral vascular disease) and found only supportive evidence when beta-blockers are administered systemically (i.e., to reduce individuals' exercise tolerance and exercise work output). These effects are not simply the result of a reduction in heart rate or blood pressure during exercise, such as ophthalmic doses may cause, but are due to a more complex alteration in energy and electrolyte metabolism [38–41]. Dickstein et al. demonstrated that timolol solution, timolol gellan, and betaxolol reduce the heart rate and systolic arterial blood pressure at baseline and during exercise, but they found no difference in work output. In particular, the effect on the heart rate was statistically more significant than the effect on systolic arterial blood pressure [53].

These data provide additional support for the concepts that heart rate and blood pressure alone do not account for differences in exercise capacity and that ophthalmically administered doses may be insufficient to affect work output, despite the statistically significant changes in hemodynamic parameters.

Asthma affects roughly 5% of the Western population. In a clinical trial where timolol drops were used in asthmatic and nonasthmatic patients, there might be an upregulation of beta-2 receptors after chronic administration, causing bronchospasm and worsening other chronic obstructive airway disease or asthma. Thus, glaucoma patients with concomitant pulmonary disease generally should not receive these agents until further data are available [4–8]. In nonasthmatic patients, neither 0.5% timolol solution nor 0.1% timolol gel has noteworthy clinical effects on respiratory function and blood pressure [42].

In addition, many other side effects have been described including depression, increased low-density cholesterol levels, hair loss, sexual impotence, fatigue, confusion, and disorientation. The systemic effects of topical beta-blockers are mainly proportional to timolol plasma levels due to the nasopharyngeal tract absorption. The association between beta-blockers and depression is largely based on published case reports and short case series, while when clinical trials and large population-based surveys of patients receiving systemic beta-blocker therapy were performed, no association was found [19].

However, when a single daily administration of 0.1% timolol gel is used, the systemic bioavailability is largely under threshold level.

In normolipidemic patients, 0.5% timolol solution seemed to lower HDL cholesterol levels by 8 to 11% with the impending increase of LDL + VLDL ratio from 10 up to 24%. This adverse effect appears to decrease when timolol in gel formulation is used, with a significant fall of value from 16 to 18% in lipidic profile formula; this potential effect may be due to the reduction of systemic absorbance [54].

### 3.1. Tolerance in Patients with OAG or OH

A prospective, randomized, investigator-masked, cross-over study investigated the circadian and blood pressure (BP) reduction obtained with timolol maleate 0.5% solution administered twice daily versus timolol 0.1% in gel-forming carbomer administered in the morning in patients with POAG. Following a baseline evaluation, patients were randomized to receive timolol 0.5% solution or timolol 0.1% gel for two months and then switched to the alternative medication for further two months. Ambulatory home BP monitoring was measured at baseline and after each treatment period, systemic and diastolic BP remained generally unaffected and the calculated diastolic ocular perfusion pressure was either unaffected. This study was undertaken because of old data that topical beta-blockers may be less efficacious during the night and may also decrease ocular perfusion due to alterations in systemic circulatory parameters [27].

In another study, the authors showed a significant difference on the drug-induced change in heart rate (in peak heart rate during the exercise test and after head-up tilt test) between treatment with 0.5% timolol gel and 0.1% timolol gel but not between 0.5% aqueous solution and 0.1% timolol gel treatments although pharmacokinetics curves were similar between the both timolol 0.5% formulations [42].

### 3.2. Tolerance in Healthy Volunteers

In a pharmacological study, heart rate at rest and during exercise was compared in 42 men (aged 55 to 65 years, mean 58.6 years) who were treated with topical instillation of 0.5% timolol solution twice a daily and 0.5% timolol gel once a daily. At the end of administration, an exercise test was performed, the clinical results showed that reducing the dose administered by half allowed to reduce significantly adverse effects. No effect on respiratory function was found in the two groups [53].

## 4. Quality of Life of Patients

### 4.1. Chronic Treatment and Ocular Surface Disease

The ocular surface system is a very dynamic structure that needs to be constantly refreshed; as a matter of fact, a lack of water, lipid, or mucie component of tear film can lead to surface inflammation and damage to cornea and conjunctiva. Ocular surface diseases are often associated with eyelid disease and particularly with meibomian gland dysfunction, and that should be investigated and treated. Unfortunately, not enough attention is paid to patients who are affected with both ocular surface disease (OSD) and glaucoma, mostly when it is necessary to prescribe a IOP-lowering therapy.

Glaucoma disease often requires a treatment for life; therefore, therapy must be effective and secure with poor side effects; otherwise, patients will discontinue eyedrops; in fact, about a quarter of patients complain side effects connected to their glaucoma therapy causing more or less 64% of reasons for nonadherence to treatment [55–59].

A study performed by 249 French ophthalmologists on 1181 patients treated with antiglaucomatous therapy demonstrated that in 40 to 54% of cases administration of eyedrops was accompanied by sensation of discomfort or pain, and 51 to 58% of patients reported at least one symptom between various instillations (Levrat et al., unpublished data). A recent retrospective survey performed from 1991 to 2003 showed that patients with severe ocular surface disease more frequently suffered also from glaucoma, with a prevalence of 67.5% and an incidence of 20.4% in patients with OSD [60].

In chronic topical treatment, the intolerance can be specifically linked to active ingredients and to preservatives. Valente and Iester outlined in a review that both components can induce intolerance [61].

It has been shown that because of the active compounds or the preservatives of the topical medication, the long-term use of antiglaucoma medications induces inflammatory ocular surface changes, causing a dry eye-like syndrome [61–68]. The toxic action of preservatives on the ocular surface has been largely demonstrated and is related to concentration, duration of use, and number of instillations [64–66]. Benzalkonium chloride (BAK), whose efficacy and toxicity are well known, is the most common preservative in antiglaucoma drugs [67], but the mechanisms of toxicity in the ocular surface are complex and not entirely understood.

Generally, it is less frequent to have an immediate allergic reaction with a timolol topic agent than with other antiglaucoma topical agents; beta-blockers instead can produce corneal hypoaesthesia and a reduction in tear secretion due to their lipophilic nature and their membrane-stabilizing activity. The fall in tear secretion can be clinically assessed with reduction in tear break-up time (BUT), leading at the end to dry eye syndrome and superficial punctuate keratitis [68].

Steuth found that the anesthetic effect of a beta-blocker applied locally to the surface of the conjunctiva and cornea could decrease lacrimal gland secretion [69] and then tear production, probably by systemic and/or local effects of beta-adrenergic receptor blockade in the lacrimal and/or accessory palpebral glands. Derous et al. demonstrated that topical beta-blockers caused thickened conjunctival epithelium, abnormal keratinization, and loss of goblet cells in the conjunctiva [70]. Herreras et al. showed that 66% of patients who used topical timolol for an average of 25 months and over had abnormal Schirmer I test and BUT [62].

Sensation of stinging, burning, sand, dryness, watering, or itching are classical examples of preservative local intolerance; also conjunctival hyperaemia and follicles are frequent clinical sign in patients using a preservative topical eye drop. Unfortunately, this type of side effect has an important consequence on patient's compliance, which is very important in long-term therapy for glaucoma; it is clear that giving a treatment with a preservative-free formula increases patient's compliance reducing signs and symptoms of local disturbance [65].

It is widely demonstrated that symptoms of topical intolerance are two or three times more frequent with ophthalmic agents containing a preservative solution than a preservative-free preparations [65]. A large European study performed on 9,658 patients treated with ophthalmic beta-blockers with or without preservative, shown that symptoms experienced by patients and clinical sign were less common when administered with unpreserved formula [71].

In patients who switched glaucoma therapy from a preservative formula to a preservative-free medication was noted a significant reduction of all ocular symptoms and signs such as ocular discomfort, sensation of foreign body, and stinging or burning or sensation of dry eyes.

Preservatives are the principal cause of ocular irritation; their effect is dose- and time-dependent, and about three-quarters of ophthalmic medication contains benzalkonium chloride (BAK) [65, 72–75].

Benzalkonium chloride is one of the most frequently used ones in ophthalmic medications, and this quaternary ammonium compound is a detergent that destroys cell membranes acting as a bactericidial agent but also damaging conjunctival and corneal surface. The detergent effect of this component causes the reduction in number of mucous cells and changes in transmembrane mucins, which can lead to dry eye syndrome. BAK at very low concentration is able to activate complement system causing the release of numerous toxic free radicals that stop cell growth, while at normal concentrations, this compound conduces to irreversible apoptosis and cell death in 15 minutes [76, 77].

The toxic action of preservatives on the ocular surface, which has been widely demonstrated, might be related to preservative concentration, duration of use, and number of instillations [64–66]. Benzalkonium chloride promotes activation of lipooxygenase and synthesis and secretion of eicosanoids, inflammatory mediators, and many cytokines such as interleukin (IL)-la, tumor necrosis factor-*α*, IL-8, and IL-10, resulting in irritation, delayed hypersensitivity, and allergic reactions [78]. Green et al. showed that BAK has a very slow turnover in the eye, being retained in ocular tissues for up to 48 hours after administration of a single drop [79]. Toxicity was delayed and prolonged, probably due to incorporation and persistence of BAK molecules in cell membranes. Three mechanisms of BAK toxicity have been described: a detergent effect, causing loss of tear film stability; direct damage to the corneal and conjunctival epithelium; and immunoallergic reaction [80]. The ocular cytotoxicity of BAK has been demonstrated in many in vitro and in vivo models [65]. Manni et al. compared patients treated with preserved timolol with patients treated with preservative-free timolol and found a significantly higher level of IL-1*β* in the preserved timolol treated group [81]. Noecker et al. showed that topical hypotonic drugs containing low preservative concentrations were associated with less inflammatory infiltrate in the rabbit conjunctiva [82].

Several clinical studies on humans confirm these laboratory results. Pisella et al. found an increase of the frequency of eye irritation in glaucoma patients who use preserved eye drops. Signs and symptoms were correlated with the number of preserved eye drops used by patients [65]. Baudouin confirmed that the frequency of dry eye-like symdrome symptoms and signs were higher in patients treated with preserved than preservative-free eye drops, and the change from preserved to preservative-free preparation was associated with a significant decrease in ocular surface irritation [83].

In patients treated for a long period with antiglaucoma topical agents, inflammatory reaction is very common with signs of macrophages infiltration over the conjunctiva and expression of antigenes which is essential in the immune response of cells [84]. In studies performed with confocal microscopy comparing preservative-free 0.1% timolol in gel and other BAK-preserved eye drops, it was demonstrated that conjunctival morphology, epithelial layer, and goblet cells profile were unaffected for preservative-free timolol formula [85].

Inflammation over conjunctiva fatally causes damages to epithelium, keratinization, and loss of mucus cell and fibrous and scar tissues, and this cascade can be avoided using a preservative-free agents. For these side effects, it is essential for glaucomatous patients to avoid conjunctiva's inflammation to give trabeculectomy a chance of success and to maintain a good quality of life for a good compliance and adherence to therapy.

Minimally invasive techniques, such as confocal microscopy, are preferable for in vivo assessment of the histopathology of many ocular surface diseases [86–89] and for investigating the toxic effects of chronic glaucoma therapy [85].

Using in vivo confocal microscopy (IVCM) (HRT II Cornea Module; Heidelberg Engineering GmbH, Heidelberg, Germany), Frezzotti et al. showed that during the 1-year follow-up, no signs of ocular surface were found in both controls and patients treated with preservative-free timolol, while BAK-preserved eye drops were associated with a lower goblet cell density and epithelial irregularity which are signs of ocular surface inflammation [85]. Furthermore, in a different study, these clinical signs due to BAK have been found just after 6 months of treatment [66]. However, these are short-term intervals when compared with the chronicity of the disease.

Overall, the findings of different studies which compared preservative to preservative-free drops seem clinically relevant considering that goblet cells are responsible for mucin production, and mucins are fundamental to guarantee the adhesion of tear film to epithelia, whose homogeneity is a sign of health [86]. The relatively small changes of IVCM are often associated to relevant changes of signs and symptoms of ocular surface disfunction [90–92], a fact that is even more relevant if we consider that patients with glaucoma are very frequently exposed to multiple treatments. As the iatrogenic damage in glaucoma is mostly due to preservatives, minimizing their administration is an impelling need as Noecker and colleagues showed in the rabbit conjunctiva [82].

In conclusion, in many clinical studies, in patients with glaucoma, the use of preserved eye drops increases the frequency of ocular surface irritation, in a dose-dependent manner [65, 82], and the switch from preserved to preservative-free formulations induces recovery from eye irritation [82] as laboratory results confirmed.

### 4.2. Patient's Compliance to Therapy

A meta-analysis study performed on 1,256 patients with OAG or OH treated either with latanoprost or timolol shown that up to 18% of patients developed iris pigmentation and conjunctival hyperaemia, these sides effects were rarer with patient treated with timolol. Another frequent side effect of prostaglandin found was eyelash hypertrichosis that can be disturbing for patients because eyelash may become longer and thicker with an unusual growth, together with a increased of inflammation stimulated by proinflammatory arachidonate cascade (e.g., uveitis, iritis, and macular oedema), less frequently [93]. However, we have to remember that timolol causes iris pigmentation in about 30 % patients after four years of treatment and could affect up to 85% of patients with mixed color irides [94].

Glaucoma patients can lose quality of life (QoL) for several reasons: the diagnosis itself, the functional loss, the inconvenience of the treatment, the side effects of the treatment, and the cost of the treatment [59]. QoL can be measured by questionnaires, but it is also dependent on subjective evaluation by the patient. In this study, we introduced a questionnaire to better evaluate the visual quality and symptoms and the quality of life. Most of the both ocular surface and general symptom questions showed a statistically significant improvement in 3 months of follow-up. Also, the revised Mills and Drance's questionnaire or Viswanathan et al.'s questionnaire was used in this study because it has been shown to be the most sensitive one in glaucomatous patients [59], but the results have been used for a different study and not presented in this manuscript.

Iester et al. showed an improvement of the ocular surface after the treatment was changed and preservative-free beta-blocker used (Table 2). Besides, in the timogel drop, there is also an artificial tear, which could help the improvement of the ocular surface both for clinical signs and symptoms (Table 2); this positive effect was seen also for both break-up time and Schirmer's test [95]. In addition to this, the preservative-free formula of 0.1% timolol was better tolerated by patients and improved the quality of life (QoL) reducing dryness, hyperaemia, follicular hyperplasia, and foreign body sensation. Considering that adherence to treatment is proportional to QoL in glaucoma, it is largely preferable in these patients to use a preservative-free formula [95]. (Table 3)

In a different study, 82% of patients were satisfied or very satisfied with timogel vs 61% with previous treatment, and the difference was statistically significant (*p* < 0.01) [96].

These data are confirmed by another following study that concluded that chronic exposure to BAK-preserved topical IOP-lowering medication was associated with adverse effects for the ocular surface; instead, a treatment with a BAK-free gel formulation with timolol 0.1% resulted in a measurable improvement in TBUT, Schirmer test result, and OSD index and in the quality of life of glaucoma patients. It has been also demonstrated that the use of preservative-free timolol 0.1% gel maintained the efficacy of IOP and reduced signs and symptoms in almost all glaucomatous patients treated with a preserved latanoprost formula with signs of local intolerance [44].

In conclusion, preservative-free timolol 0.1 treatment maintained IOP at the same level of the other beta-blockers, but it was better tolerated in patients having signs or symptoms while on preserved beta-blockers. Preservative-free treatment improved the quality of life reducing dryness, hyperaemia, follicular hyperplasia, and foreign body sensation, probably for the lack of BAK and the presence of an artificial tear in the medication bottle. The once-daily dosing improves compliance and adherence.

## Figures and Tables

**Table 1:** Therapeutic efficacy of 0.1% timolol gel.

Ref.	No. of patients	Methods	IOP changes (%)	IOP change (mmHg)	Conclusion
22	210	1st line compared to 0.5% timolol solution	−27% after 12 weeks (*p* < 0.001)	−6.3/−7	No difference between treatment (*p*=0.19)
32	175 (350 eyes)	1st line preservative-free 0.1% timolol gel vs 0.1% timogel gel with preservative	−31% after 12 weeks	−5.63/ −5.63	Comparable in both groups (NS)
33	68 (110 eyes)	1st line or replacement for 0.5% timolol solution or combination with latanoprost	−27% at 6 month additional drop −13% total drop −38%	−6.3 −2.6 −11, 3	*p* > 0.001 *p* < 0.001 *p*=0.12
34	111	1st line (53 pts) or replacement for a beta-blocker (58 pts)	−25% at 12 weeks additional drop −13%	−6 −3.41	*p* < 0.001 *p* < 0.001
35	86 (119 eyes)	1st line (53 pts) or replacement for a poorly tolerated or ineffective beta-blocker (33 pts)	−25% at 3 months additional fall −11% after 3 months	−9.1 −4.5	Comparable efficacy to timolol 0.5%
36	30 (54 eyes)	Replacement for latanosprost due to poor compliance	−30% on latanoprost −25% on timolol gel	16.15 –16.5	Comparable efficacy *p*=0.53
37	55	1st line—comparison of 0.1% timolol gel and solution			IOP reduction 1 to 2 mmHg greater with the gel formulation (*p* < 0.05)

Ref. = reference; No. = number; IOP = intraocular pressure; *p* = *p* value.

**Table 2:** Comparison of the data between baseline and the follow-up by using ANOVA test and the Bonferroni post hoc test.

	Baseline	1 month	3 months	Baseline vs. 1 month	1 month vs. 3 months	Baseline vs. 3 months
	Mean (SD)	Mean (SD)	Mean (SD)	*p* value	*p* value	*p* value
Eyelid erythema	0.46 (0.82)	0.23 (0.55)	0.13 (0.37)	<0.001	<0.001	<0.001
Conjunctival hyperaemia	0.97 (0.94)	0.58 (0.64)	0.33 (0.52)	<0.001	<0.001	<0.001
Follicular hyperplasia	0.36 (0.62)	0.16 (0.40)	0.08 (0.31)	<0.001	<0.001	<0.001
Beak-up time (sec)	9.82 (0.31)	10.9 (3.24)	11.5 (3.38)	<0.001	<0.001	<0.001
Schirmer test (min)	13.46 (6.28)	14.72 (6.44)	15.41 (6.32)	<0.001	<0.001	<0.001

From [95].

**Table 3:** Beta-blocker and quality of life.

Questionnaire	ANOVA with Bonferroni post hoc	
Ocular surface symptoms	Baseline vs after 1 month	After 1 month vs after 3 months
*Have you experienced any of the following during the last week:*	*p* value	*p* value
(1) Eyes that are sensitive to light?	0.003	0.002
(2) Foreign body sensation?	<0.001	0.880
(3) Painful eye?	<0.001	<0.001
(4) Blurred vision?	0.007	<0.001
(5) Poor vision?	0.208	<0.001
(6) Ocular redness?	<0.001	<0.001
General symptoms		
*Have you experienced any of the following during the last week:*		
(7) Breathlessness at rest	0.045	<0.001
(8) Breathlessness after exercise	0.083	<0.001
(9) Fatigue, faintness	0.001	0.059
(10) Slow heart beat	0.321	<0.001
(11) Insomnia	0.038	0.279
(12) Headache	0.251	<0.001
*Did you have any problems with your eyes that limited you in performing any of the following during the last week:*		
(13) Reading?	<0.001	<0.001
(14) Driving at night?	0.001	0.001
(15) Working with a computer?	0.001	<0.001
(16) Watching TV?	0.001	<0.001
*Have your eyes felt uncomfortable in any of the following situations during the last week:*		
(17) Windy conditions ?	<0.001	<0.001
(18) Place or areas with low humidity (very dry)?	<0.001	<0.001
(19) Areas that are air-conditioned ?	<0.001	<0.001
*During the last week*:		
(20) How do you assess the intensity of the ocular surface symptoms?	<0.001	<0.001
(21) Were you satisfied with the treatment?	<0.001	<0.001
(22) How many times did not you instill the eye drop?	0.006	0.001
(23) How was your quality of life related to treatment?	<0.001	<0.001

From [95].
